# Acceptability, feasibility, fidelity and quality implementation of the culturally adapted version of the Social Competence Promotion Program among Young Adolescents (“Mi Mejor Plan”) to prevent substance use among adolescents in Chile: a pilot randomized control study

**DOI:** 10.1186/s12889-025-23033-3

**Published:** 2025-05-20

**Authors:** Saray Ramirez, Natalia Ríos, Cristian A. Rojas-Barahona, Marcela Cárcamo, Alejandro Sepulveda-Pañaloza, Ricardo Araya, Jorge Gaete

**Affiliations:** 1 Research Center for Student Mental Health (ISME), School of Education, Faculty of Social Sciences, Universidad de los Andes, Santiago, Chile; 2 ANID, Millennium Science Initiative Program, Millennium Nucleus to Improve the Mental Health of Adolescents and Youths, Imhay, Santiago, Chile; 3 Faculty of Psychology, Universidad de Talca, Talca, Chile; 4 ANID, Millenium Nucleus for the Sciences of Learning, Santiago, Chile; 5 Department of Epidemiology and Health Studies, Faculty of Medicine, Universidad de los Andes, Santiago, Chile; 6 Department of Health Service and Population Research, King’s College London, London, UK

**Keywords:** Adolescents, Substance Abuse, Prevention, Acceptability, Feasibility, Fidelity

## Abstract

**Introduction:**

Substance use among adolescents is a public health problem. We culturally adapted The Social Competence Promotion Program for Young Adolescents (SCPP-YA) program to the school context in Chile (henceforth “Mi Mejor Plan or MMP”) and assessed the acceptability, feasibility, fidelity, and quality of the implementation among 6^th^ graders. We also explored the efficacy of the program in improving individual protective factors and reducing risk factors and substance use.

**Methods:**

Cluster randomized controlled trial conducted in Chile. The schools were randomly assigned to one of two conditions in a 1:1 ratio: 1) the "MMP" intervention group, and 2) the Control group. The program consisted of a 16-h class-based curriculum promoting social problem-solving skills delivered by a trained facilitator. Primary outcomes were acceptability, feasibility, fidelity, and quality of the implementation using detailed reports of facilitators and from observers of the performance of the facilitators in vivo. Additionally, we explored the efficacy of the intervention on secondary outcomes: 30-day prevalence of tobacco, alcohol, and cannabis use and individual risk and protective factors promoted by MMP. We performed an intention-to-treat analysis using mixed models, taking into account the hierarchical nature of the data.

**Results:**

Seven hundred sixty-five 6th graders from 11 schools were enrolled (one school dropped out after the randomization); 608 were analyzed at baseline, and 538 were analyzed post-intervention. 52.5% were male, and the average age was 11.3 in both groups. All 16 sessions were implemented, and students’ attendance at each session ranged from 83.8% to 92.4%. The program was generally well-received, with up to 91.3% of students rating acceptability positively. Facilitators and observers reported high adherence to the contents of the program in most sessions. Protective factors, such as negative beliefs about tobacco and alcohol, increased significantly in the IG. Still, there were no significant changes in substance use, risk factors, emotion regulation, or school membership.

**Conclusions:**

The MMP program was well accepted, and we achieved high levels of implementation and fidelity. The program improved some individual protective factors, such as negative beliefs about tobacco and alcohol, with no changes in substance use among adolescents.

**Trial registration:**

ClinicalTrials.gov, number NCT04236947; registration date: 17/01/2020.

**Supplementary Information:**

The online version contains supplementary material available at 10.1186/s12889-025-23033-3.

## Introduction

Globally, mental and substance use disorders are topics of particular concern regarding the global burden of disease among children and youth, accounting for 25% of all years lived with a disability (YLD) and 5.7% of disability-adjusted life years (DALYs) [1]. In addition, the use of alcohol, cannabis, and nicotine affects brain development during adolescence [2], increasing the risk of depression [3], anxiety [4], cognitive problems [5], psychosis [6], and suicidality [7]. These acute and chronic alterations have several consequences for future development, such as work opportunities and health and legal problems [8].

In Chile, the last available national study conducted in 2021 showed that 11.1% of adolescents smoked tobacco in the last month, 24.0% drank alcohol, and 11.2% used cannabis [9], making Chile one of the top countries in the prevalence of substance abuse among secondary students in the Americas [10]. Although this prevalence has been somewhat reduced recently, they are still alarming, especially considering the increase in crimes related to drug trafficking in Chile [11]. The Chilean government has implemented different initiatives in the last decades, such as creating the Explicit Health Guarantee for the Harmful Use and Dependence of Alcohol and Other Drugs for people under 20 years old, which grants benefits for the treatment and post-intervention of alcohol and drug abuse. It has covered 26,571 cases up to March 2023 [12]. Second, the implementation of a manualized school-based universal prevention program called “Continuo Preventivo” which includes four to six lessons per academic year between preschool and 12 th grade, since 2009 [13]. The effectiveness of this intervention has not been tested using a randomized controlled study [14]. Schools are a significant social context for adolescents. Here, students interact with peers and teachers, and it is a space for learning academic subjects and life skills [15]; and several studies have proved the importance and usefulness of implementing preventive measures in schools [15]. However, it is equally important to provide evidence of the acceptance, feasibility, and preventive impact of these interventions to avoid potential negative results [16, 17], especially if they are not well-informed by theory [18] or studied using rigorous methodology [19].

Several international substance use prevention programs have been successfully implemented in the school context with positive results. First, The Life Skills Training Program is a multi-component preventive intervention for students to develop substance resistance skills by learning self-management strategies, establishing healthy relationships, and engaging in responsible decision-making [20]. Another such program is The Lion’s Quest, Skills for Adolescence Program, which aims to develop socio-emotional competencies in students from preschool to 12 th grade and includes a Substance Prevention Module [21]. Finally, the Unplugged program, based on the theory of cognitive social influence, promotes intrapersonal skills (critical thinking versus normative beliefs) and interpersonal skills (assertiveness and skills of how to resist peer pressure) while also providing information on the different substances [22, 23].

The programs mentioned above have a complex theory of change, including several theoretical approaches, from Social Learning Theory to Normative beliefs theory and promoting the development of different intrapersonal and interpersonal skills [20–22], which sometimes makes it difficult to determine the actual mediators of the effectiveness of the intervention. Additionally, the payment of a usage license and the need for a costly certified training program reduces the possibility of scaling up the program if the results show it to be effective. Most of these programs can be implemented for students of different ages, making it also difficult for schools to choose the most convenient time for the intervention. Finally, all school-based interventions tested in other cultural contexts require an adaptation and a close collaboration with original developers. Roger Weissberg’s New Haven Adolescent Socio-Emotional Development Program [24], also known as the Social Competence Promotion Program for Young Adolescents (SCPP-YA) [25], has been evaluated and found effective in substance use prevention among adolescents. It uses a clear theory of change, no license costs are involved, there is a clear target age group, which makes it ideal for exploring the potential benefits and mediating variables of effectiveness; and finally, our research team had a strong relationship with the developer which helped to adapt the intervention appropriately.

The Social Competence Promotion Program for Young Adolescents (SCPP-YA) [25], was developed in 1990 and successfully implemented in numerous schools in the USA, especially in areas with greater economic vulnerability. Its first results showed that 6 th graders receiving the program, relative to controls, improved their strategies for resisting peer pressure and coping with stress and developed more negative attitudes about substances [26]. Later, a randomized controlled trial showed that the program had several beneficial effects for young teens: it allowed them to acquire problem-solving skills, improved their social relationships with peers, reduced their behavioral problems and alcohol consumption, and decreased the development of criminal behaviors [27]. Regarding problem-solving capacity, students who participated in this program improved the quantity and quality of their problem-solving choices compared to the control group [27]. The program also increased positive engagement with peers [25].

The program SCPP-YA is a manualized universal prevention intervention for 6 th graders. Its curriculum has a core module called Problem-solving Skills and other problem-oriented modules such as the Substance Use Prevention module. The first module has 27 sessions of 45 min, each focused on practicing several problem-solving skills based on six steps: (1) Stop, calm down, and think before you act; (2) Say the problem and how you feel; (3) Think of a positive goal; (4) Think of lots of solutions; (5) Think ahead to the consequences; and (6) Go ahead and try the best plan using interactive methodologies. The Substance Use Prevention module has nine sessions of 45 min, each centered on learning how to prevent substance use and how the skills learned in the problem-solving module can be applied to prevent substance use.

The SCPP-YA program aims to reduce substance use by targeting a range of cognitive, emotional, and social mediators, which has been evaluated in other preventive interventions among adolescents, and they are important. The program includes activities to address these mediators. At its core, the program strengthens problem-solving skills [28] and self-regulation [29] through a structured six-step model, which enhances emotional regulation, cognitive planning, and goal setting. Additionally, the Substance Use Prevention module seeks to modify both positive and negative beliefs about substance use—including beliefs related to tobacco, alcohol, and marijuana—by informed decision-making. By reducing positive beliefs [30] and reinforcing negative beliefs [31] about substance use, the program aims to shift students’ attitudes in a protective direction. The intervention further seeks to increase risk perception [31] related to substance use and strengthen protective factors such as self-efficacy and future orientation. Moreover, the program trains refusal skills, by improving students’ ability to manage peer pressure and make autonomous decisions in high-risk situations [32]. Together, these mediators are theorized to reduce intentions for future substance use, serving as the mechanisms through which the program ultimately promotes healthier decision-making and prevents the initiation of risky behaviors.

This program was the starting point of a significant educational movement: implementing interventions to promote social and emotional learning (SEL) skills. Roger Weissberg was one of the founders of Collaborative for Academic, Social, and Emotional Learning (CASEL), a Chicago-based nonprofit organization supporting SEL implementation since 1994 [33].

This program has never been studied in Latin American countries. Our research team foresaw the potential benefits of the cultural adaptation of this program. It started to work with Roger Weissberg to make this program more suitable for the Chilean educational reality while maintaining the core components of the intervention. The result was a newly adapted program called “Mi Mejor Plan” (MMP), which included a core problem-solving module of 11 sessions and a substance use prevention module of 5 lessons for 6 th graders, which can be implemented in one academic year, and three booster sessions in the following year.

Cultural adaptation of school-based substance use prevention programs is a critical component in enhancing their relevance, acceptability, and overall effectiveness among adolescent populations. Adolescents’ attitudes, beliefs, and behaviors related to substance use are deeply embedded within their sociocultural environments, which shape normative expectations, communication styles, and perceptions of risk. Implementing prevention programs without adequate cultural adaptation may limit their impact, as they may fail to resonate with students’ lived experiences or address context-specific risk and protective factors. By aligning the program’s content, language, and delivery methods with the cultural norms and values of the target population, cultural adaptation can improve student engagement, facilitate comprehension of key messages, and strengthen the intervention’s behavioral impact. Furthermore, culturally responsive interventions are more likely to promote equitable outcomes across diverse groups, thereby supporting broader public health goals in adolescent substance use prevention.

Conducting a pilot study is a critical step in the implementation of evidence-based interventions, particularly when adapting programs to new cultural or educational contexts. A pilot allows for the systematic assessment of key implementation outcomes—namely acceptability, feasibility, and fidelity—which are essential for determining whether a program can be effectively delivered in real-world settings [34]. Assessing acceptability helps identify how well the intervention is received by students, educators, and other stakeholders, informing potential refinements to increase engagement and relevance. Evaluating feasibility ensures that the program’s structure, duration, and resource demands align with the constraints and capacities of the school environment. Monitoring fidelity verifies that the program is being implemented as designed, which is crucial for maintaining the integrity of its theoretical foundations and expected outcomes. Together, these indicators provide valuable information that can guide necessary adaptations, support scale-up efforts, and increase the likelihood of achieving meaningful and sustained impacts on adolescent substance use prevention [34].

Given the exploratory nature of this study and its relatively small sample size, the pilot study serves a crucial role in generating preliminary evidence regarding the potential impact of the intervention [35]. While statistical power may be limited, pilot studies are not primarily designed to detect statistically significant effects but rather to provide effect size estimates that inform the practical or clinical relevance of the outcomes. Reporting these effect sizes allows readers and future researchers to evaluate the magnitude and direction of the observed effects, even in the absence of statistical significance. Moreover, these estimates offer valuable guidance for the planning of larger, adequately powered trials by informing sample size calculations and refining the selection of outcome measures. Emphasizing effect sizes in this context aligns with best practices in prevention science, where early-stage studies contribute to the evidence base by identifying promising intervention components and informing subsequent phases of research.

Therefore, the aims of this pilot study were: 1) to assess the acceptability and feasibility of “Mi Mejor Plan” in the school context in Chile among 6^th^ graders; 2) to assess the fidelity and quality of the implementation; and 3) to explore the efficacy of the program comparing self-reported substance use and risk and protective factors of 6^th^ graders participating in the MMP program and control schools immediately after the end of the intervention, controlling by baseline assessment. The impact and acceptability of the booster sessions and follow-up assessments are not part of this article.

## Materials and methods

### Study design, participants, procedure, and ethical considerations

This is a cluster randomized controlled trial, parallel-group type, with two arms: (1) MMP group and (2) control group, with the standard school prevention curricula. Schools were the unit of allocation, and individual participants were the unit of analysis. The trial registration identifier is NCT04236947 in Clinical Trials [ClinicalTrials.gov].

Inclusion criteria of schools:

1. Schools located in Region Metropolitana (RM), Chile.
2. Schools of Primary Education (1 st grade to 8 th grade). The MMP curriculum was designed to be implemented in 6 th grade.
3. Mixed-sex schools.
4. Schools with at least two classes in the 6 th grade.
5. Schools with at least 30 students per class
6. Schools with vulnerability (≥ 50%). We used the School Vulnerability Index – National System of Equality Allocation (IVE-SINAE) to state the degree of vulnerability. This index is the proportion of students with high vulnerability in a given school. This index considers the following socioeconomic variables to group the schools: mother’s educational level, father’s educational level, and total monthly household income, among others [36].

Exclusion criteria of schools:

1. Four or more classes in 6 th grade. This criterion was considered for economic and practical reasons because the larger the school, the more expensive the implementation and supervision.
2. Implementing other manualized substance use prevention programs. However, schools may implement preventive lessons in the Orientation curriculum (see below).

**Fig. 1:**
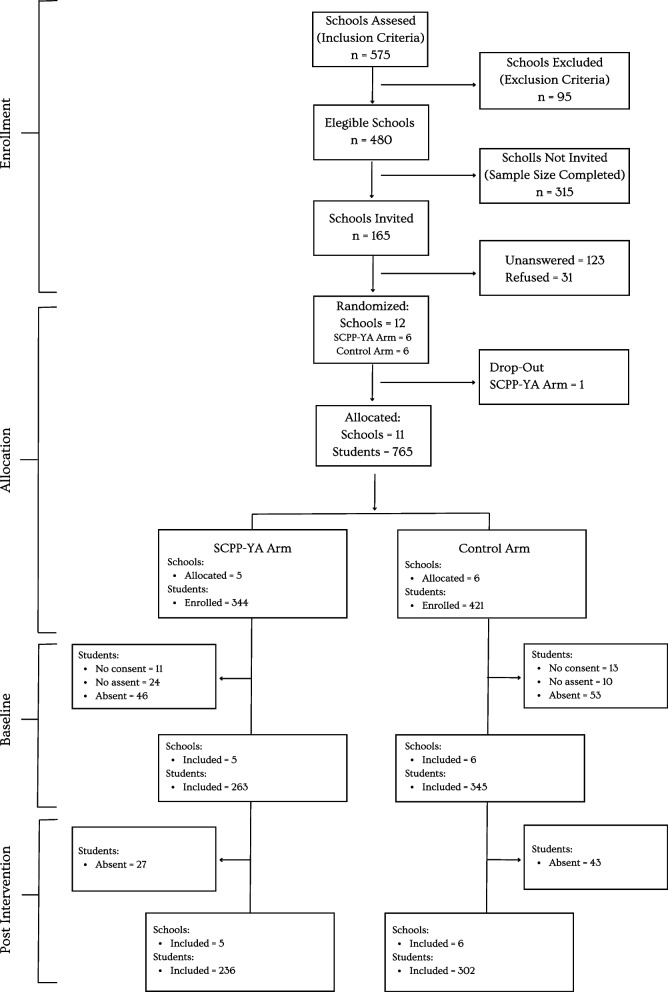
Recruitment; baseline; and post-intervention of schools and students

The recruitment for this study started in November 2019; however, in December 2019, the SARS-CoV-2 pandemic started, and the Chilean Minister of Health and the Minister of Education implemented sanitary measures that kept schools closed until October 2021. We stopped the recruitment and restarted at the end of 2021 to be ready to start the implementation in March 2022. To recruit schools for the present study, we implemented the strategy of First Come, First Served (FCFS), which implies that schools were invited till the sample size was completed because of the exploratory nature of the project and limited resources. Out of 575 Schools in RM, 480 schools were eligible for the study. We sent invitations to schools until we reached the sample size planned. Finally, we completed 165 invitations, and school authorities were informed about the study aims, methods, and assessments. Twelve schools agreed to participate and signed a consent. Randomization took place before the baseline assessment by an independent statistician (1:1) to assign schools to either MMP or control groups using a computer-generated randomization sequence. After randomization, all schools were informed of their allocation; at this time, one school from the intervention arm withdrew, saying that they did not have time for the implementation; this resulted in having five schools in the intervention arm and six schools in the control arm. Then, an informed and written consent form was sent to the parents or main caregivers for signature. Finally, students also provided informed and written assent. The total number of students enrolled in the schools was 765, and after signed consent by caregivers and assented by students, 608 (79.5%) adolescents answered the baseline assessment questionnaire (April 2022). In the post-intervention assessment (October 2022), 538 students answered the questionnaire. See the flowchart in Fig. 1.

Regarding ethical considerations, data was collected following the Declaration of Helsinki [37], with the approval of the ethics committee of the Universidad de los Andes (CEC201983, November 5 th, 2019). The students and their parents or main caregivers received no reimbursement for participating in this study. Students or parents/main caregivers could request the withdrawal from the study at any time throughout the project. Confidentiality was granted for all assessments, and an independent statistician handled and coded the information.

### Sample size

Since this is a pilot study, it is not appropriate to calculate a sample size for establishing the effectiveness of the intervention [38]. However, we have calculated a suitable number of adolescents for this study. According to recommendations for feasibility studies, a minimum of 30 participants per arm is helpful to estimate the parameters for future sample size calculations [39]. In addition, we wanted to ensure some variability, so we proposed to include three schools per arm, with three grades (Year 6, 7, and 8), with at least 60 students per grade in each arm. However, to test the efficacy or the effectiveness of school-based preventive interventions, it is necessary to work with a large sample size [40], so no definitive conclusions can be drawn from exploring the efficacy presented in this study.

### Intervention and control group

#### Intervention group

##### Adaptation

The original SCPP-YA was developed by Roger Weissberg and collaborators in 1990 and first implemented in New Haven (USA) [25]; they gave authorization to the research team to adapt the program to the Chilean context and rename it “Mi Mejor Plan” (MMP). First, a translation was done by a bilingual professional translator and revised by two research assistants; afterward, the research team performed a linguistic adaptation. We interviewed potential stakeholders and schoolteachers to understand the potential obstacles to implementing the whole program in Chilean educational settings. Those interviews provided valuable information about the current preventive activities in the school and the time arranged for those activities during the academic year. For instance, all schools in Chile have a class for preventive lessons every week of the academic year. This class is called “Orientation”. The number of potential “Orientation” classes is 38 for the whole academic year [41]; however, schools usually use at least half of the classes to organize students for several other non-academic activities such as school trips, sports events, and conflict resolution meetings. Therefore, the availability for implementing preventive programs is less than 20 classes. Additionally, each session should last under 45 min to use the time provided for preventive lessons within the school curriculum. Therefore, a content adaptation was needed to have a shortened program version to fit schools’ calendars during one academic year.

The program curriculum has two modules: the first one is called the Problem-solving Skills module, and the second one is called the Substance Use Prevention Module. The first module was shortened to 11 sessions from the original 27 sessions of 45 min. Each session focused on practicing several problem-solving skills based on six steps of problem-solving strategy [25]: (1) Stop, calm down, and think before you act; (2) Say the problem and how you feel; (3) Think of a positive goal; (4) Think of lots of solutions; (5) Think ahead to the consequences; and (6) Go ahead and try the best plan using interactive methodologies. The second module was shortened to five sessions from the original nine sessions of 45 min, each centered on learning how to prevent substance use, how to use the skills learned in problem-solving to improve performance on social problems, and how to deal with peer influence. The research team analyzed the superficial and core components of the original program [42] and the theory of change presented in the Introduction. We carefully culturally adapted all superficial aspects of the program (e.g., pictures, posters, characters) and kept the sessions'core components. However, we faced a difficult challenge in the adaptation process. Adapting the program requires conducting culturally relevant changes while keeping the core elements [42, 43]. The original program had 27 sessions (problem-solving module) plus nine sessions for the substance use module. In this challenging decision, most of the sessions that we reduced were those dedicated to practicing the skills, trying to condense the core components of the program (the 6-step problem-solving method) into 11 sessions, always providing interactive activities to practice all steps of the problem-solving method. The same was true for the sessions for the substance use model. In addition, we carefully kept the sequential approach of the problem-solving structure presented in the original manual. In both modules, even though we intended to keep the core components, we may have compromised the effect of the intervention through at least two mechanisms. On the one hand, reducing the exposure time needed to adopt a skill students (by reducing the practice sessions) may have negatively impacted the skill acquisition. On the other hand, altering the whole structure of the program, the mediators of the intervention, even though they may act independently of each other, the combined indirect effects separately for each mediator may differ from the overall natural indirect effect if there are additive interactions between the mediators'effects on the outcome [44].

The new 16-session program was culturally appropriate and increased the feasibility of implementation in Chilean settings. Each session was organized into three sections, each with specific activities: opening, central, and closure. Details of the session selection process can be found in Supplementary Material 1.

##### Implementation

The intervention group implemented the adapted version of the SCPP-YA program called “Mi Mejor Plan (MMP)” during the academic year within school hours, usually in the “Orientation” weekly classes. The program’s curriculum was delivered by a trained facilitator (member of the research team) and the school teacher (who was also trained). The program includes the facilitator´s manual and the student´s workbook.

#### Control group

The control group received standard school drug prevention curricula (“Treatment-As-Usual,” TAU), which is usually a non-manualized intervention. The standard curriculum in the “Orientation” class may include teaching on health promotion and preventive messages for substance use.

### Measures

#### Primary outcomes

##### Acceptability, feasibility, and fidelity

**Acceptability**

Students provided information regarding acceptability using a self-reported survey that included four sections related to the perception and their experience in the sessions: (1)“General acceptability” (4 items); (2) “Implementation & material acceptability"(5 items); (3) “Helpfulness of MMP program” (7 items); and (4) “Satisfaction with MMP program” (4 items). All items were responded with a Likert scale (1 = “Strongly disagree/Strongly dissatisfied” to 5 = “Strongly agree/Strongly satisfied”).

**Feasibility**

The feasibility of the recruitment process was measured using a registry of consent, assent, absence, and questionnaire response rates. We also registered the degree of attendance of students by session.

**Fidelity and quality of implementation**

We used a questionnaire that included three dimensions of fidelity [45]: (1) adherence, (2) pedagogy, and (3) logistics, answered by the facilitator after the delivered sessions. Additionally, an observer attended sessions in the schools when they were delivered, registered, and rated in written form questions regarding two dimensions of fidelity (adherence and pedagogy). It was expected that observers rated at least 25% of all delivered sessions.

Adherence refers to how much the activities described in the manual were fully implemented. In the case of facilitators, they reported if they implemented the activities in their entirety, and if they could not implement all activities, and provided the reasons for doing so. For the latter, we used the following options: Lack of time and difficulty in group management. In the case of observers, adherence was assessed in more detail, providing a rating of all sections of each session: opening, central, and closure activities. Observers provided the assessment score in six possibilities. Briefly, 0% (Not Started), 20% (Barely Started), 40% (Partially Complete), 60% (Moderately Complete), 80% (Almost Complete), and 100% (Fully Complete). We also assess the degree of knowledge of the manual demonstrated by the facilitator during sessions. It followed the following scoring: 0% (No Knowledge); 20% (Basic Initial Knowledge); 40% (Partial Knowledge); 60% (Moderate Knowledge); 80% (Advanced Knowledge); and 100% (Expert Knowledge).

Pedagogy refers to strategies to facilitate delivery quality, such as physical space preparation, improving participation and group dynamics, and the perception of students´ responsiveness (e.g., attention and participation during the sessions). These strategies were assessed by facilitators after each session, using a 5-point scale from 1 (Never) to 5 (Always). Additionally, we evaluated group management by observers using a scale based on the following scoring in a 6-level: 0% (No Control); 20% (Minimal Control); 40% (Partial Control); 60% (Moderate Control); 80% (Strong Control); and 100% (Mastery of Group Management).

Logistics refers to the duration of each session and the total number of sessions implemented.

Other detailed aspects of fidelity and quality of implementation, such as adherence by each facilitator and school, are presented in the Supplementary Material 3.

#### Secondary outcomes

1. The European Drug Addiction Prevention Trial Questionnaire (EU-Dap) [46]. EU-Dap is a self-reported questionnaire that aims to screen substance use and assess risk factors (positive beliefs about tobacco, positive beliefs about alcohol, positive beliefs about cannabis, normative beliefs, and future substance use) and protective factors (negative beliefs about tobacco, negative beliefs about alcohol, negative beliefs about cannabis, risk perception, and refusal skills) among adolescents. It has 45 multi-item questions and focuses especially on tobacco, alcohol, and cannabis use. The EU-Dap instrument has been validated in Chile; all subscales had acceptable internal reliability (omega ≥ 0.65) [47].
2. The Social Problem-Solving Inventory-Revised (SPSI-RS) [48]. SPSI-RS consists of 25 items measuring five subscales that evaluate two dimensions of positive (Positive Problem Orientation and Rational Problem-Solving) and negative (Negative Problem Orientation, Impulsive/Careless Style, and Avoidance Style) problem-solving strategies. Each subscale has five items, answered on a 5-choice Likert-like scale, in which the subjects indicate to what extent the proposed coping strategy applies to their characteristic way of dealing with the problems of daily life (0 = Very untrue, 1 = Somewhat untrue, 2 = Neutral, 3 = Somewhat true, and 4 = Very true). There is also a total problem-solving score, which includes the 25 items, where the higher the score, the better the performance in the problem-solving strategies. In this study, we used the total score in the analysis, which demanded that the score of all negative scales be reversed. It has acceptable internal reliability (Cronbach alpha > 0.75) [49].
3. The Emotion Regulation Questionnaire for Children and Adolescents (ERQ-CA) [50]. Emotional regulation is the ability to control and manage one’s emotions and appears to be an essential factor in social adjustment. This instrument has ten items organized into two scales: Cognitive Reappraisal (six items), which consists of redefining a potential emotion-eliciting situation in a way that its emotional impact is changed, and Expressive Suppression (four items), encompassing a style that consists of the inhibition of ongoing emotion expressive behavior. For each statement, the answers are on a five-point Likert scale quantifying the agreement with the behavior described (1 = strongly disagree, 2 = disagree, 3 = half and half, 4 = agree, 5 = strongly agree). It has been validated in Chile and showed acceptable internal reliability (Cronbach alpha ≥ 0.75) [51].
4. The Psychological Sense of School Membership Scale (PSSM) [52]. The sense of school membership refers to the student’s perceptions of the respect and acceptance that teachers and other students show them and their sense of belonging. It has 13 items (Chilean version) and is a single dimension. Our research team validated the scale in Chile in early adolescents, showing a high internal reliability (Cronbach’s alpha 0.92) [53].

### Statistical analyses

The Consolidated Standards of Reporting Trials (CONSORT) statement was followed for reporting the results of this study [54].

For primary outcomes, students'acceptability of the program was described using frequencies of the degree of agreement with several statements. The feasibility of the recruitment, assessment, and intervention implementation was described using frequencies. For the fidelity and quality of the implementation (adherence, pedagogy, and logistics), we present the frequencies, proportions, and means of scores for different items of surveys completed by facilitators and observers.

For secondary outcomes, all schools and participants were included in the analysis using an intention-to-treat approach [54]. First, we used descriptive statistics to assess balance across arms at baseline. We analyzed each outcome measure using two different 2-level mixed models; the first model (Model 1) accounts for fixed effects for baseline scores, group effect (intervention vs control), time (baseline, endpoint). The second model (Model 2) accounts for the same fixed effects as Model 1 but adds sex and age as covariates. Every model included a random effect for school and participants (allowing correlations within schools and repeated measures within participants). For binary outcomes, we used logistic mixed-effects model analysis to compare the intervention and control groups from baseline to post-intervention, and then we calculated odds ratios. For continuous outcomes, we used linear mixed-effects model analysis to compare the intervention and control groups from baseline to post-intervention. We calculated adjusted mean differences with 95% Cis and Cohen´s *d* effect size. All analyses were performed using STATA 17.

## Results

### Descriptive features at baseline

A total of 608 students were included in the baseline of the study. A 52.5% of students were male in both intervention and control groups, and their mean age was 11.3 in both groups. Substance use was slightly higher in the intervention group, especially in the category of cannabis use in the last month. Regarding the risk and protective factors for substance use assessed with the EU-Dap questionnaire, all risk factors, such as “positive beliefs about tobacco”, were slightly higher in the intervention group, and all protective factors, such as “negative beliefs about tobacco”, were slightly lower in the intervention group. Emotion regulation strategies were similar in both groups. Social problem-solving skills were poorer in the intervention group. School membership score was also lower in the intervention group. See Table 1.

**Table 1:** Baseline characteristics

	Intervention (MMP)			Control		
Variables	n	% or mean	[95% CI] or (SD)	n	% or mean	[95% CI] or (SD)
Sex						
Female	125	47.5	[41.5–53.6]	164	47.5	[42.3–52.8]
Male	138	52.5	[46.4–58.5]	181	52.5	[47.2–57.7]
Age	263	11.3	(0.6)	345	11.3	(0.5)
Type of School dependency						
Subsidized	181	68.8	[62.9–74.2]	345	100	-
Public	82	31.2	[25.8–37.1]	-	-	-
IVE-SINAE	263	77.8	(12.4)	345	78.4	(7.5)
Substance use						
Tobacco in the last month	5	1.9	[0.8–4.5]	4	1.2	[0.4–3.1]
Alcohol in the last month	18	6.8	[4.3–10-6]	18	5.2	[3.3–8.1]
Drunkenness in the last month	3	1.1	[0.4–3.5]	2	0.6	[0.1–2.3]
Marijuana in the last month	3	1.1	[0.4–3.5]	-	-	-
EU-Dap						
Positive beliefs about tobacco	242	7.6	(3.2)	313	7.3	(3.2)
Negative beliefs about tobacco	249	9.8	(3.4)	326	10.1	(3.3)
Positive beliefs about alcohol	235	9.7	(4.3)	308	9.3	(4.3)
Negative beliefs about alcohol	247	13.2	(4.6)	323	13.4	(4.7)
Positive beliefs about marijuana	242	7.5	(3.7)	324	7.3	(3.6)
Negative beliefs about marijuana	242	17.0	(6.1)	322	17.4	(5.7)
Future substance use	251	7.9	(3.9)	330	7.1	(3.5)
Normative beliefs friends	253	4.6	(1.4)	336	4.2	(0.9)
Normative beliefs other adolescents	254	6.7	(3.4)	323	6.5	(3.3)
Risk perception	238	19.6	(4.3)	319	19.9	(4.1)
Refusal skills	253	10.7	(2.2)	330	10.9	(1.7)
ERQ-CA						
Cognitive Reappraisal	234	19.3	(6.3)	306	19.8	(6.4)
Expressive Suppression	238	13.0	(4.4)	309	12.7	(4.3)
SPSI-RS	206	49.4	(11.4)	262	51.9	(11.9)
PSSM	221	44.1	(12.7)	283	45.6	(12.0)

*n* number of students, *CI* Confidence interval, *SD* Standard deviation

### Primary outcomes

#### Acceptability

In general, the program had good acceptability by the students. All aspects assessed in the “General acceptability” and “Implementation and material acceptability” sections were considered highly positive, up to 91.3% (“Agree/Strongly agree that the Teacher and the Facilitator get along”). Regarding the “Helpfulness” section, students reported a high sense of helpfulness, especially that “The program is useful to learn about the dangers of drugs” (84.6% reported Agree/Strongly agree). Finally, 77.5% of students reported being satisfied/strongly satisfied with the program. See Table 2.

**Table 2:** Students´ acceptability of the program

	Strongly disagree (1)		Disagree (2)		Nor agree nor disagree (3)		Agree (4)		Strongly agree (5)		
General Acceptability	n	%	n	%	n	%	n	%	n	%	Total Mean (SD)
I liked the sessions	10	5.8	6	3.5	35	20.3	62	36.1	59	34.3	3.8 (1.0)
I learned	8	4.7	7	4.1	42	24.7	59	34.7	54	31.8	3.8 (1.0)
I liked the activities	6	3.5	10	5.9	30	17.5	47	27.5	78	45.6	4.0 (1.0)
I think the program helped me	10	5.8	13	7.6	41	23.9	65	38.0	42	24.6	3.6 (1.1)
Implementation & material acceptability											
Teacher and Facilitator get along	2	1.1	1	0.6	12	6.9	53	30.6	105	60.7	4.4 (0.7)
Teacher participates and collaborates during the sessions	3	1.7	4	2.3	22	12.8	58	33.7	85	49.4	4.2 (0.8)
I like the type of activities used in the sessions	7	4.1	9	5.2	29	16.8	48	27.8	80	46.2	4.0 (1.0)
The workbook used helps me in during the sessions	11	6.4	12	6.9	29	16.9	74	43.0	46	26.7	3.7 (1.1)
I like the design and format of the workbook	14	8.1	14	8.1	34	19.8	48	27.9	62	36.1	3.7 (1.2)
Helpfulness: “The program helped…											
… to improve school environment specially in our class	10	5.9	18	10.5	39	22.8	48	28.1	56	32.8	3.7 (1.1)
… me learn how to relate to others better	11	6.4	13	7.6	45	26.3	58	33.9	44	25.7	3.6 (1.1)
… me manage my emotions better	17	10.0	15	8.8	44	25.9	47	27.7	47	27.7	3.5 (1.2)
… me to learn about the dangers of drugs	3	1.8	3	1.8	20	11.8	43	25.4	100	59.2	4.3 (0.8)
… all classmates to relate better with each other	18	10.6	22	12.9	46	27.1	47	27.7	37	21.8	3.3 (1.2)
… me today to have more skills to avoid using tobacco, alcohol or drugs	12	7.0	13	7.6	27	15.8	42	24.6	77	45.0	3.9 (1.2)
… me to have more skills to avoid using tobacco, alcohol, or drugs in the future	9	5.3	8	4.7	31	18.1	39	22.8	84	49.1	4.0 (1.1)
Satisfaction: “Thinking about the"MMP"Program in general,…	Strongly dissatisfied (1)		Dissatisfied (2)		Nor satisfied nor dissatisfied (3)		Satisfied (4)		Strongly satisfied (5)		
… how satisfied are you with the facilitator?	3	1.8	2	1.2	18	10.5	46	26.9	102	59.7	4.4 (0.8)
… how satisfied are you with the length of the program?	16	9.4	12	7.1	35	20.6	62	36.5	45	26.5	3.6 (1.2)
… how satisfied are you with the activities?	9	5.3	6	3.6	21	12.4	49	29.0	84	49.7	4.1 (1.1)
… how satisfied are you with the program in general?	4	2.4	8	4.7	26	15.4	49	29.0	82	48.5	4.1 (1.0)

*n* number of students, *SD* Standard deviation

#### Feasibility

Out of 575 Schools in RM, 480 schools were eligible for the study. 165 were invited to participate in the project, and school authorities were informed about the study aims, methods, and assessments. 123 schools never answered our invitation, and 31 refused to participate, mainly for not accepting the possibility of being allocated to the control group. Finally, 12 agreed to participate and signed a consent. After randomization, but before the baseline assessment, one school in the intervention group withdrew from the study, saying they no longer had the time to implement the sessions. See Flowchart in Fig. 1.

Regarding the students, the questionnaire response rate at baseline concerning school enrollment was 76.5% and 81.9%, and 78.9% and 84.5% concerning the consent in the intervention and control groups, respectively. These figures at post-intervention assessment were 68.6% and 71.7%, and 70.8% and 74.0%, respectively. See Table 3.

**Table 3:** Students´ response rate at baseline and post-intervention

	Intervention (MMP)		Control		Total	
	n	%	n	%	n	%
Students enrolled	344	100	421	100	765	100
Student consent	333	96.8	408	96.9	741	96.8
Students who answered questionnaire at baseline in relation to the enrollment	263	76.5	345	81.9	608	79.4
Students who answered questionnaire at baseline in relation to the consent	263	78.9	345	84.5	608	82.0
Students who answered questionnaire at post-intervention in relation to the enrollment	236	68.6	302	71.7	538	70.3
Students who answered questionnaire at post-intervention in relation to the consent	236	70.8	302	74.0	538	72.6

*n* number of students

The level of attendance at the sessions can be seen in Table 4. By session, the lowest attendance was in session 2 (84.4%), and the highest was in session 13 (92.4%). By school, attendance rate ranged from 83.8% to 91.8%. See Table 4.

**Table 4:** Students´ attendance

	School 1 (n total = 36)		School 2 (n total = 61)		School 3 (n total = 72)		School 4 (n total = 48)		School 5 (n total = 46)		Total by session (n total = 263)	
	n	%	n	%	n	%	n	%	n	%	n	%
Session 1	32	88.8	51	83.6	66	91.7	41	85.4	41	89.1	231	87.8
Session 2	31	86.1	51	83.6	63	87.5	39	81.3	38	82.6	222	84.4
Session 3	29	80.6	55	90.2	64	88.9	46	95.8	38	82.6	232	88.2
Session 4	32	88.8	53	86.9	66	91.7	46	95.8	36	78.2	233	88.6
Session 5	34	94.4	56	91.8	66	91.7	43	89.6	39	84.7	238	90.5
Session 6	35	97.2	60	98.4	64	88.9	43	89.6	38	82.6	240	91.3
Session 7	28	77.7	59	96.7	69	95.8	43	89.6	35	76.0	234	89.0
Session 8	30	83.3	55	90.2	70	97.2	44	91.6	36	78.2	235	89.4
Session 9	32	88.8	55	90.2	69	95.8	47	97.9	37	80.4	240	91.3
Session 10	29	80.6	53	86.9	67	93.1	38	79.2	38	82.6	225	85.6
Session 11	29	80.6	59	96.7	60	83.3	43	89.6	38	82.6	229	87.1
Session 12	31	86.1	59	96.7	66	91.7	45	93.8	41	89.1	242	92.0
Session 13	33	91.6	56	91.8	69	95.8	44	91.6	41	89.1	243	92.4
Session 14	32	88.8	58	95.1	63	87.5	44	91.6	41	89.1	238	90.5
Session 15	32	88.8	56	91.8	67	93.1	44	91.6	41	89.1	240	91.3
Session 16	30	83.3	51	83.6	69	95.8	43	89.6	39	84.7	232	88.2
Total mean	31	86.6	55	90.9	66	91.8	43	90.2	39	83.8	235	89.4

*n* number of students, *n total* number of students in the intervention group (baseline)

#### Fidelity and quality of implementation

The following information regarding facilitators’ reports can be seen in 5a-5 d, and the observer´s reports can be seen in Tables 5, 6, 7 and 8.

**Table 5:** Program adherence reported by facilitators and observers

Facilitator’s self-report	*N* = 160^a^	%
Did I implement the session in its entirety?		
Yes	99	61.9
No, because lack of time	48	30.0
No, because the classroom management was difficult	13	8.1
Observer’s report	*N* = 52^b^	Mean of %
Sessions implemented in its entirety	14	26.9
Completion of opening activities^c^	52	94.6
Completion of central activities^c^	52	78.5
Completion of closure activities^c^	52	81.2
Degree of knowledge of the manual^d^	52	98.5

^a^Number of sessions with Facilitator´s reports. All potential sessions were implemented and reported

^b^Number of sessions with Observer´s reports. The observer attended and rated the implementation by facilitators of 52 sessions (32.5% of all sessions)

^c^The observer rated completion of each section; 0% (Not started) to 100% (Fully complete)

^d^The observer rated degree of knowledge of the manual; 0% (No Knowledge) to 100% (Expert Knowledge)

**Table 6:** Program pedagogy reported by facilitators and observers

**Facilitator’s self-report**	Always		Often		Sometimes		Rarely		Never	
**Quality of delivery** (*n* = 160)^a^	n	%	n	%	n	%	n	%	n	%
How often did I prepare the space for the session?	95	59.4	50	31.3	14	8.8	1	0.6	0	0
How often did I use strategies to improve students’ participation?	107	66.9	35	21.9	17	10.6	1	0.6	0	0
How often did I use strategies to improve group dynamics?	109	68.1	34	21.2	16	10.0	1	0.6	0	0
**Participant responsiveness** (*n* = 160)^a^										
How often did the students pay attention?	72	45.0	43	26.9	33	20.6	7	4.4	5	3.1
How often did the students participate?	87	54.4	45	28.1	19	11.9	7	4.4	2	1.3
**Observer’s report** (*n* = 52)^b^	n				Mean of %					
Group management^c^	52				90.4					

^a^Number of sessions with Facilitator´s reports. All potential sessions were implemented and reported

^b^Number of sessions with Observer´s reports. The observer attended and rated the implementation by facilitators of 52 sessions (32.5% of all sessions)

^c^The observer rated group management; 0% (No Control) to 100% (Mastery of Group Management)

**Table 7:** Program logistics reported by facilitators

**Facilitator’s self-report**^**a**^	N^b^	%
**Duration of each session**		
I met the designated time	141	88.1
I outrun the designated time	10	6.3
I did not fill the designated time	9	5.6

^a^We only registered this information in the questionnaire answered by the facilitator

^b^Number of sessions with Facilitator´s reports. All potential sessions were implemented and reported

**Table 8:** Program logistics, number of sessions implemented by the facilitator

	Sessions^a^																	
	1	2	3	4	5	6	7	8	9	10	11	12	13	14	15	16	Total (*n*^b^ = 160)	%
Facilitator 1	8	7	7	4	5	5	5	5	5	5	5	5	5	5	5	5	86	53.8
Facilitator 2	2	2	1	2	2	2	2	2	2	2	2	2	2	2	2	2	31	19.4
Facilitator 3	0	1	1	1	2	2	1	2	2	2	2	2	2	2	2	2	26	16.3
Facilitator 4	0	0	0	0	0	0	0	0	1	1	1	1	1	1	1	1	8	5.0
Facilitator 5	0	0	1	3	0	0	1	0	0	0	0	0	0	0	0	0	5	3.1
Facilitator 6	0	0	0	0	1	1	1	1	0	0	0	0	0	0	0	0	4	2.5

^a^Number of sessions. All potential sessions were implemented and reported

^b^*n* number of sessions

##### Adherence

Six facilitators implemented sessions of the program. Facilitators´ self-reports were obtained for 160 sessions (all sessions implemented). Facilitators reported that all activities were conducted in most of them (*n* = 139, 61.9%). However, 61 sessions were performed partially because of a lack of time (30.0%) or difficulties in group management due to the students´ behavior (8.1%).

Regarding observer´s reports, 52 sessions were assessed in the field (32%). The assessment showed that adherence was high for all sections of sessions: opening (94.6%), central activities (77.2%), and closure activity (80.9%). The degree of knowledge demonstrated by facilitators was close to “expert knowledge” (98.5%). For further information see Supplementary Material 2.

Regarding the performance of each facilitator and school, see Supplementary Material 3.

##### Pedagogy

Most of the facilitators reported that they often or always prepared the space for the session (90.7%) and used strategies to improve the student’s participation (88.8%) and group dynamics (89.3%). Participants had high levels of attention (71.9%) and participation (82.5%).

Group management was assessed by the observer close to “Mastery of group management” (90.4%).

##### Logistics

Regarding the duration of each session, 88.1% of the facilitators used the designated time, 6.3% outrun the designated time, and 5.6% used less of the designated time.

All 16 sessions (100%) were implemented. Most sessions were implemented by one facilitator (53.8%).

### Secondary outcomes

Even though substance use variables had higher odds in the intervention group than the control group, the results are not statistically significant. See Table 9.

**Table 9:** Substance use

	Model 1	Model 2
Variables	OR (95% CI)	OR (95% CI)
Substance use		
Tobacco in the last month	1.34 (0.25–7.17)	1.21 (0.21–6.99)
Alcohol in the last month	1.73 (0.85–3.48)	1.69 (0.81–3.51)
Drunkenness in the last month	1.74 (0.29–10.29)	1.75 (0.28–11.13)
Marijuana in the last month	1.35 (0.24–7.56)	1.33 (0.24–7.48)

*OR* Odds ratio, *CI* Confidence Interval, *Model 1* adjusted for baseline outcome measures, *Model 2* adjusted for sex, age and baseline outcome measures

None of the risk factors were significantly reduced with the program. Regarding the protective factors, negative beliefs about tobacco (Cohen´s *d* = 0.30) and alcohol (Cohen´s *d* = 0.30) were both significantly increased in the intervention group compared with the control group.

The intervention did not improve emotion regulation and problem-solving strategies. Finally, school membership was also not improved in the IG. See Table 10

**Table 10:** Risk and protective factors

	Model 1	Model 2	
Variables	aMD (95% CI)	aMD (95% CI)	Cohen´s *d* effect size
EU-Dap			
Risk factors			
Positive beliefs tobacco	0.62 (−0.13–1.42)	0.62 (−0.14–1.43)	0.19
Positive beliefs alcohol	0.75 (−0.27–1.78)	0.75 (−0.24–1.75)	0.18
Positive beliefs marijuana	0.82 (−0.06–1.71)	0.82 (−0.05–1.69)	0.22
Normative beliefs friends	−0.03 (−0.47–0.41)	−0.03 (−0.46–0.39)	−0.02
Normative beliefs other adolescents	−0.08 (−0.60–0.44)	−0.09 (−0.62–0.44)	−0.02
Future substance use	0.22 (−0.60–1.04)	0.21 (−0.60–1.03)	0.06
Protective factors			
Negative beliefs tobacco	0.98 (0.01–2.07)	**0.98 (0.01–1.95)**	0.30
Negative beliefs alcohol	**1.32 (0.19–2.46)**	**1.32 (0.18–2.46)**	0.30
Negative beliefs marijuana	1.22 (−0.54–2.9)	1.18 (−0.52–2.88)	0.21
Risk perception	0.57 (−0.37–1.51)	0.54 (−0.43–1.50)	0.14
Refusal skills	−0.35 (−0.88–0.18)	−0.35 (−0.89–0.18)	−0.17
ERQ-CA			
Cognitive Reappraisal	−0.04 (−1.48–1.39)	−0.04 (−1.46–1.38)	−0.01
Expressive Suppression	0.68 (−0.20–1.56)	0.64 (−0.24–1.52)	0.14
SPSI-RS	−1.25 (−2.95–0.46)	−1.00 (−2.74–0.73)	−0.07
PSSM	−0.03 (−2.22–2.16)	0.01 (−2.21–2.24)	0.00

*p* < 0.05 are in bold

*aMD* adjusted mean difference, *CI* Confidence Interval, *Model 1* adjusted for baseline outcome measures, *Model 2* adjusted for sex, age and baseline outcome measures

## Discussion

This is the first study exploring the acceptability, feasibility, and fidelity of the adapted version of The Social Competence Promotion Program among Young Adolescents (SCPP-YA), under the name of “Mi Mejor Plan” (MMP) in Latin America. This study also explored the efficacy of the intervention in reducing substance use and risk and promoting protective factors. All sessions were implemented, and the facilitators and observers reported high fidelity and quality of the intervention. The intervention was well received by students, and most of them said that they learned about the risk of using substances of abuse and thought that the program gave them skills to reduce using drugs in the future. Although there were no differences in the 30-day prevalence of substance use, it seems that the program increased critical protective factors such as negative beliefs about tobacco and alcohol.

Although there are some preventive programs showing effectiveness in high-income countries, such as School Health and Alcohol Harm Reduction Project (SHAHRP) in Australia [55], Life Skills Training (LST) program in the USA [56–58], and Unplugged in seven European countries [59], fewer interventions have been rigorously tested in Latin America. For instance, Unplugged has been studied in Brazil [60] and Chile [23] with promising results. Our research team already adapted Unplugged when we decided to adapt SCPP-YA to Chile, and this work guided the adaption process in this study. Although Unplugged includes some content promoting social problem-solving skills [58, 61], SCPP-YA uses this strategy as the main drive to generate behavioral changes among early adolescents. For our team, it was important to test another intervention on substance use prevention and give adolescents, schools, and policymakers a wider range of options when deciding which intervention best suits the school culture.

Regarding feasibility, our team implemented all 16 sessions in the intervention group. Some experiences have found that trained school teachers do not perform well on fidelity and implementation [47]. In contrast, other studies have found better implementation quality when teachers delivered the program compared with external facilitators [62]. While others have found no differences between teachers and external specialists in student outcomes or implementation quality [63]. We decided to hire and train external facilitators, either free-lance teachers or psychologists, to reduce the risk of failure in the intervention delivery. This consideration was based on Chilean teachers'well-known high workload in day-to-day activities [64], making it challenging to include the training, delivery, and supervision during the program's implementation in their routine. However, we decided to train external facilitators in the same training sessions along with school teachers to provide good knowledge of the program and get to know each other to become close collaborators during classroom sessions. In this regard, the highest scores reported by students in the acceptability survey were the items “Teacher and Facilitator get along” (4.4, SD = 0.7) and “How satisfied are you with the facilitator” (4.4, SD = 0.8). Therefore, they perceived the collaboration and relationship between teachers and facilitators to be good. We considered this good relationship a potential factor explaining the high degree of implementation.

The program was well accepted by adolescents with a high degree of satisfaction, and they liked the activities. The program included interactive activities, role-playing, and games to increase participation. Some authors have recommended that school-based preventive interventions should include these kinds of practices to promote active student learning [65, 66]. We may be confident that the changes appealed to the adolescents because the program was well accepted. Adolescents also could identify the aims of the program because they reported that the program helped them to know about drugs and to learn the skills needed to reduce drugs in the future. These results are consistent with the findings of increasing some of the protective factors promoted by the program.

Adapting the program was challenging, requiring culturally relevant changes while preserving core elements [42, 43]. As we have explained in the Methods regarding the adaptation process, reducing the practice sessions may have negatively affected skill development, knowing that practice is important. Additionally, affecting the whole structure of the original program may have cut out some indirect effects of the mediators'interactions [44]. Knowing that practice is essential, we decided to provide more opportunities to practice the skills and designed three booster sessions, which were implemented in the following year. The whole 2-year MMP program results will be presented in future publications.

Finally, the exploratory results of the efficacy showed that the MMP program increased negative beliefs about tobacco and alcohol in the intervention group compared to the control group. A recent study of the effectiveness of Unplugged in Nigeria showed that the intervention significantly reduced the prevalence of recent alcohol use in the intervention compared to control pupils. Negative beliefs about substance use were one of the mediators of Unplugged's effect on alcohol use. Therefore, there is some evidence that these protective factors could benefit students in the future. On the other hand, there was no evidence of efficacy in reducing any of the measured substance use. They may be disappointing results, but these are exploratory. This was a pilot study with the primary objective of describing the acceptability and feasibility of the implementation of MMP without the statistical power to test either the efficacy or the effectiveness of this intervention. Additionally, the students in the intervention group had poorer results in substance use and on the risk and protective variables measured at baseline. This may reflect that these students were coming to the study with more difficulties than the control group. A larger randomized controlled study may help to reduce the imbalance between groups. Finally, the post-intervention assessment happened right after the end of the 16 th session. Some of the promoted skills may unfold over time, underscoring the necessity for ongoing practice and reinforcement [67], so follow-up assessments are valuable for exploring this hypothesis and seeing if the expected preventive effect appears with time.

### Limitations

One key limitation was the reliance on self-reported data, which may be subject to social desirability bias. Additionally, the intervention took place after the pandemic when schools were re-installing normal academic activities with many disruptions in functioning. Chile was the Organization for Economic Co-operation and Development (OECD) member country that kept schools closed for the longest time during the pandemic. The country closed schools for 147 school days in 2020 and 112 in 2021, totaling 259. Chile was the only member nation of the OECD that exceeded 250 days of school closures [68]. Even though the after-pandemic was a difficult time for schools, the implementation of the program reached high levels of feasibility. As we already mentioned, some imbalances regarding substance use and risk and protective factors and differences in school characteristics, such as size and administrative structure, also arose by chance between groups.

Additionally, when comparing the groups, the control group was considered as Treatment-As-Usual because we did not interfere with the content of the “Orientation” class implemented in all schools in Chile. A limitation in this regard can be related to the lack of information the research team had on the content, material, and pedagogy used in these classes and whether they were or were not related to substance use prevention. Another limitation is related to the representative of our results. The schools included in this study were located in highly deprived areas and served low-income families. This is the case for 89% of schools in Chile [69], so they represent a large population in Chile. However, these results cannot be extrapolated to middle- to high-income families. An additional limitation for a larger expansion of the MMP program is related to the fact that we used external trained facilitators to deliver the intervention. This decision helped us ensure high fidelity; however, it limited cost-effectiveness and widespread scale-up potential due to the added costs and efforts. Despite these limitations, the study’s primary focus on acceptability and feasibility yielded valuable insights for planning larger-scale evaluations of the intervention. Finally, even though we recognized the importance of assessing acceptability among external facilitators and schoolteachers, we did not plan to systematically assess it because of the workload in their teaching activities, making it difficult for them to answer an acceptability survey. We plan to include their perspectives in future phases of the study.

This is a limitation for future implementation, especially if we want to make this kind of intervention sustainable in time. Future research should include easy options to assess this information and make this process more participatory.

## Conclusions

This is the first study of the SCPP-YA (MMP) adaptation to prevent substance use among early adolescents in Latin America. The program was well accepted, and we achieved high levels of implementation and fidelity. The program improved some individual protective factors, such as negative beliefs about tobacco and alcohol. However, preliminary results on the efficacy of reduction of substance use showed no effect of the intervention. Future studies should explore the long-term effect of the intervention and the impact of the three booster sessions implemented in the following year. Moreover, these findings offer important contributions to the field of prevention science in Chile by highlighting key areas for intervention within school settings. The results underscore the need for culturally relevant, evidence-based approaches that address both individual and contextual factors influencing substance use. From a policy perspective, there is still a challenge in how school-based prevention strategies can be integrated into preventive education into the national curriculum, alongside systematic schoolteacher training in these matters. Solving these issues could enhance the sustainability and effectiveness of prevention efforts, contributing to a more comprehensive and proactive public health response.

## Supplementary Information

Supplementary Material 1.

Supplementary Material 2.

Supplementary Material 3.

## Data Availability

The datasets used and/or analysed during the current study are available from the corresponding author on reasonable request.

## Abbreviations

SCPP-YA: Social Competence Promotion Program for Young Adolescents
YLD: Years lived with a disability
DALYs: Disability-adjusted life years
RM: Region Metropolitana
FCFS: First Come, First Served
ICC: Intraclass correlation coefficient
TAU: Treatment-As-Usual
EU-Dap: The European Drug Addiction Prevention Trial Questionnaire
SPSI-RS: The Social Problem-Solving Inventory-Revised
ERQ-CA: The Emotion Regulation Questionnaire for Children and Adolescents
PSSM: The Psychological Sense of School Membership Scale
CONSORT: Consolidated Standards of Reporting Trials

## References

[1] Erskine HE, Moffitt TE, Copeland WE, Costello EJ, Ferrari AJ, Patton G, et al. A heavy burden on young minds: the global burden of mental and substance use disorders in children and youth. Psychol Med. 2015;45(7):1551–63. 25534496 10.1017/S0033291714002888 PMC5922255

[2] Thorpe HHA, Hamidullah S, Jenkins BW, Khokhar JY. Adolescent neurodevelopment and substance use: Receptor expression and behavioral consequences. Pharmacol Ther. 2020;206:107431. 31706976 10.1016/j.pharmthera.2019.107431

[3] Calarco CA, Lobo MK. Depression and substance use disorders: Clinical comorbidity and shared neurobiology. Int Rev Neurobiol. 2021;157:245–309. 33648671 10.1016/bs.irn.2020.09.004

[4] Williams GC, Patte KA, Ferro MA, Leatherdale ST. Substance use classes and symptoms of anxiety and depression among Canadian secondary school students. Health Promot Chronic Dis Prev Can. 2021;41(5):153–64. 33982903 10.24095/hpcdp.41.5.02

[5] Meruelo AD, Castro N, Cota CI, Tapert SF. Cannabis and alcohol use, and the developing brain. Behav Brain Res. 2017;325(Pt A):44–50. 28223098 10.1016/j.bbr.2017.02.025 PMC5406224

[6] García Álvarez L, Gomar JJ, García-Portilla MP, Bobes J. Cannabis use and cognitive impairment in schizophrenia and first-episode psychosis. Adicciones. 2019;31(2):89–94. 31017245 10.20882/adicciones.1328

[7] Rioux C, Huet AS, Castellanos-Ryan N, Fortier L, Le Blanc M, Hamaoui S, et al. Substance use disorders and suicidality in youth: a systematic review and meta-analysis with a focus on the direction of the association. PLoS One. 2021;16(8):e0255799. 34358273 10.1371/journal.pone.0255799 PMC8345848

[8] Degenhardt L, Stockings E, Patton G, Hall WD, Lynskey M. The increasing global health priority of substance use in young people. Lancet Psychiatry. 2016;3(3):251–64. 26905480 10.1016/S2215-0366(15)00508-8

[9] Observatorio Chileno de Drogas. Décimo Cuarto Estudio Nacional de Drogas en Población Escolar de Chile 2021 8º Básico a 4º Medio. Santiago: Servicio Nacional para la Prevención y Rehabilitación del Consumo de Drogas y Alcohol (SENDA). Ministerio del Interior y Seguridad Pública; 2023. Available from: https://www.senda.gob.cl/wp-content/uploads/2023/07/14_EstudioDrogas_Poblacion_Escolar.pdf.

[10] Hynes M, Clarke P, Cumsille F, Araneda-Ferrer J, Ahumada G. Informe sobre el consumo de drogas en las Américas 2019. Washington DC: Organización de los Estados Americanos; 2019.

[11] De La Costa F. INFORME: ANÁLISIS DEL AUMENTO DE LA DELINCUENCIA EN CHILE Y CORRELACIÓN CON LA SITUACIÓN MACRO Y MICROECONÓMICA DEL PAÍS. Valparaiso: Senado de Chile; 2024.

[12] Superintendencia de Salud. Estadística Trimestral de Casos GES (AUGE) de Fonasa y Sistema Isapre a marzo 2023. Available from: http://www.supersalud.gob.cl/documentacion/666/w3-article-23615.html.

[13] Ministerio del Interior y Seguridad Pública. Programa Continuo Preventivo. Available from: https://www.senda.gob.cl/prevencion/iniciativas/prevencion-escolar/programa-continuo-preventivo/.

[14] Hariton E, Locascio JJ. Randomised controlled trials - the gold standard for effectiveness research: study design: randomised controlled trials. BJOG. 2018;125(13):1716. 29916205 10.1111/1471-0528.15199 PMC6235704

[15] Jiménez AK, Mella FR, Gálvez-Nieto JL. School climate and substance use in a sample of Chilean adolescents. Rev. psicodidáct. 2023;28(2):164–72.

[16] Sloboda Z, Stephens RC, Stephens PC, Grey SF, Teasdale B, Hawthorne RD, et al. The Adolescent Substance Abuse Prevention Study: a randomized field trial of a universal substance abuse prevention program. Drug Alcohol Depend. 2009;102(1–3):1–10. 19332365 10.1016/j.drugalcdep.2009.01.015

[17] Hawthorne G. Life Education’s failure to face the facts. Addiction. 1995;90(10):1404–6. 8616470 10.1111/j.1360-0443.1995.tb03560.x

[18] Renaud L, O’Loughlin J, Déry V. The St-Louis du Parc Heart Health Project: a critical analysis of the reverse effects on smoking. Tob Control. 2003;12(3):302–9. 12958393 10.1136/tc.12.3.302 PMC1747750

[19] Baron G, Perrodeau E, Boutron I, Ravaud P. Reporting of analyses from randomized controlled trials with multiple arms: a systematic review. BMC Med. 2013;11:84. 23531230 10.1186/1741-7015-11-84 PMC3621416

[20] Botvin GJ, Griffin KW. Life skills training as a primary prevention approach for adolescent drug abuse and other problem behaviors. Int J Emerg Ment Health. 2002;4(1):41–7. 12014292

[21] Gol-Guven M. The effectiveness of the Lions Quest Program: Skills for Growing on school climate, students’ behaviors, perceptions of school, and conflict resolution skills. Eur Early Child Educ Res J. 2017;25(4):575–94.

[22] Faggiano F, Galanti MR, Bohrn K, Burkhart G, Vigna-Taglianti F, Cuomo L, et al. The effectiveness of a school-based substance abuse prevention program: EU-Dap cluster randomised controlled trial. Prev Med. 2008;47(5):537–43. 18657569 10.1016/j.ypmed.2008.06.018

[23] Salgado G, Gaete J, Gana S, Valenzuela D, Araya R. Acceptability, feasibility and fidelity of the culturally adapted version of Unplugged (“Yo Se Lo Que Quiero”), a substance use preventive program among adolescents in Chile: a pilot randomized controlled study. BMC Public Health. 2024;24(1):2026. 39075465 10.1186/s12889-024-19499-2 PMC11285342

[24] Weissberg R, Caplan M, Bennetto L, Jackson A. The New Haven Social Development Program: Sixth-grade social problem-solving model. New Haven, CT: Yale University; 1990.

[25] Weissberg RP, Barton HA, Shriver TP. The Social-Competence Promotion Program for Young Adolescents. In: Albee GW, Gullotta TP, editors. Primary Prevention Works. California, US: SAGE Publications, Inc.; 1997.

[26] Elias MJ, Weissberg RP. School-based social competence promotion as a primary prevention strategy: a tale of two projects. Prev Hum Serv. 1990;7(1):177–200.

[27] Caplan M, Weissberg RP, Grober JS, Sivo PJ, Grady K, Jacoby C. Social competence promotion with inner-city and suburban young adolescents: effects on social adjustment and alcohol use. J Consult Clin Psychol. 1992;60(1):56–63. 1556286 10.1037//0022-006x.60.1.56

[28] Winters KC, Lee S, Botzet A, Fahnhorst T, Nicholson A. One-year outcomes and mediators of a brief intervention for drug abusing adolescents. Psychol Addict Behav. 2014;28(2):464–74. 24955669 10.1037/a0035041 PMC4075470

[29] Pentz MA, Riggs NR, Warren CM. Improving substance use prevention efforts with executive function training. Drug Alcohol Depend. 2016;163:S54–9. 27306732 10.1016/j.drugalcdep.2016.03.001 PMC12341295

[30] Giannotta F, Vigna-Taglianti F, Rosaria Galanti M, Scatigna M, Faggiano F. Short-term mediating factors of a school-based intervention to prevent youth substance use in Europe. J Adolesc Health. 2014;54(5):565–73. 24332392 10.1016/j.jadohealth.2013.10.009

[31] Vigna-Taglianti F, Mehanović E, Alesina M, Damjanović L, Ibanga A, Pwajok J, et al. Effects of the “Unplugged” school-based substance use prevention program in Nigeria: a cluster randomized controlled trial. Drug Alcohol Depend. 2021;228:108966. 34509736 10.1016/j.drugalcdep.2021.108966

[32] Wynn SR, Schulenberg J, Kloska DD, Laetz VB. The mediating influence of refusal skills in preventing adolescent alcohol misuse. J Sch Health. 1997;67(9):390–5. 9471092 10.1111/j.1746-1561.1997.tb07183.x

[33] Weissberg RP. Promoting the social and emotional learning of millions of school children. Perspect Psychol Sci. 2019;14(1):65–9. 30799753 10.1177/1745691618817756

[34] Schultes M-T. An introduction to implementation evaluation of school-based interventions. European Journal of Developmental Psychology. 2023;20(1):189–201.

[35] Thabane L, Ma J, Chu R, Cheng J, Ismaila A, Rios LP, et al. A tutorial on pilot studies: the what, why and how. BMC Med Res Methodol. 2010;10(1):1. 20053272 10.1186/1471-2288-10-1 PMC2824145

[36] Gobierno de Chile Agencia de Calidad de la Educación. Metodología de Construcción de Grupos Socioeconómicos 2013. Available from: http://archivos.agenciaeducacion.cl/Metodologia_de_Construccion_de_Grupos_Socioeconomicos_Simce_2013.pdf.

[37] Lewis JA, Jonsson B, Kreutz G, Sampaio C, van Zwieten-Boot B. Placebo-controlled trials and the Declaration of Helsinki. Lancet. 2002;359(9314):1337–40. 11965296 10.1016/S0140-6736(02)08277-6

[38] Arain M, Campbell MJ, Cooper CL, Lancaster GA. What is a pilot or feasibility study? A review of current practice and editorial policy. BMC Med Res Methodol. 2010;10(1):67. 20637084 10.1186/1471-2288-10-67 PMC2912920

[39] Lancaster GA, Dodd S, Williamson PR. Design and analysis of pilot studies: recommendations for good practice. J Eval Clin Pract. 2004;10(2):307–12. 15189396 10.1111/j..2002.384.doc.x

[40] Gruijters SLK, Peters GY. Meaningful change definitions: sample size planning for experimental intervention research. Psychol Health. 2022;37(1):1–16. 33210937 10.1080/08870446.2020.1841762

[41] Ministerio de Educación Gobierno de Chile. Orientación Programa de Estudio para Sexto Año Básico. Santiago Chile, 2013. Available from: https://bibliotecadigital.mineduc.cl/bitstream/handle/20.500.12365/663/MONO-166.pdf?sequence=1&isAllowed=y.

[42] Sundell K, Ferrer-Wreder L, Fraser MW. Going Global: A Model for Evaluating Empirically Supported Family-Based Interventions in New Contexts. Eval Health Prof. 2014;37(2):203–30. 23291390 10.1177/0163278712469813

[43] Castro FG, Barrera M Jr, Holleran Steiker LK. Issues and challenges in the design of culturally adapted evidence-based interventions. Annu Rev Clin Psychol. 2010;6:213–39. 20192800 10.1146/annurev-clinpsy-033109-132032 PMC4262835

[44] Taguri M, Featherstone J, Cheng J. Causal mediation analysis with multiple causally non-ordered mediators. Stat Methods Med Res. 2018;27(1):3–19. 26596350 10.1177/0962280215615899 PMC5698181

[45] Macklem GL. Preventive Mental Health at School: Evidence-Based Services for Students. New York: Springer; 2013.

[46] Faggiano F, Vigna-Taglianti F, Burkhart G, Bohrn K, Cuomo L, Gregori D, et al. The effectiveness of a school-based substance abuse prevention program: 18-month follow-up of the EU-Dap cluster randomized controlled trial. Drug Alcohol Depend. 2010;108(1–2):56–64. 20080363 10.1016/j.drugalcdep.2009.11.018

[47] Ramírez S, Gana S, Godoy MI, Valenzuela D, Araya R, Gaete J. Validation of the European Drug Addiction Prevention Trial Questionnaire (EU-Dap) for substance use screening and to assess risk and protective factors among early adolescents in Chile. PLoS One. 2021;16(10):e0258288. 34634082 10.1371/journal.pone.0258288 PMC8504767

[48] D’zurilla TJ, Nezu AM. Development and preliminary evaluation of the Social Problem-Solving Inventory. Psychol Assess: J Consult Clin Psychol. 1990;2(2):156.

[49] Sorsdahl K, Stein DJ, Myers B. Psychometric properties of the social problem solving inventory-revised short-form in a South African population. Int J Psychol. 2017;52(2):154–62. 26249118 10.1002/ijop.12192

[50] Gullone E, Taffe J. The Emotion Regulation Questionnaire for Children and Adolescents (ERQ-CA): a psychometric evaluation. Psychol Assess. 2012;24(2):409–17. 22023559 10.1037/a0025777

[51] Villacura-Herrera C, Gaete J, Andaur J, et al. Evidence for validity, reliability and measurement invariance of the emotion regulation questionnaire for children and adolescents (ERQ-CA) in secondary students from Chile. Curr Psychol. 2023;42:27771–82.

[52] Goodenow C. The psychological sense of school membership among adolescents: scale development and educational correlates. Psychol Sch. 1993;30(1):79–90.

[53] Gaete J, Montero-Marin J, Rojas-Barahona CA, Olivares E, Araya R. Validation of the Spanish Version of the Psychological Sense of School Membership (PSSM) Scale in Chilean Adolescents and Its Association with School-Related Outcomes and Substance Use. Front Psychol. 2016;7:1901. 27999554 10.3389/fpsyg.2016.01901 PMC5138456

[54] Butcher NJ, Monsour A, Mew EJ, Chan AW, Moher D, Mayo-Wilson E, et al. Guidelines for Reporting Outcomes in Trial Reports: The CONSORT-Outcomes 2022 Extension. JAMA. 2022;328(22):2252–64. 36511921 10.1001/jama.2022.21022

[55] McBride N, Farringdon F, Midford R, Meuleners L, Phillips M. Harm minimization in school drug education: final results of the School Health and Alcohol Harm Reduction Project (SHAHRP). Addiction. 2004;99(3):278–91. 14982537 10.1111/j.1360-0443.2003.00620.x

[56] Botvin GJ, Griffin KW, Diaz T, Ifill-Williams M. Drug abuse prevention among minority adolescents: posttest and one-year follow-up of a school-based preventive intervention. Prev Sci. 2001;2:1–13. 11519371 10.1023/a:1010025311161

[57] Botvin GJ, Griffin KW, Diaz T, Ifill-Williams M. Preventing binge drinking during early adolescence: one-and two-year follow-up of a school-based preventive intervention. Psychol Addict Behav. 2001;15(4):360. 11767269 10.1037//0893-164x.15.4.360

[58] Griffin KW, Botvin GJ, Nichols TR, Doyle MM. Effectiveness of a universal drug abuse prevention approach for youth at high risk for substance use initiation. Prev Med. 2003;36(1):1–7. 12473419 10.1006/pmed.2002.1133

[59] Caria MP, Faggiano F, Bellocco R, Galanti MR. Effects of a school-based prevention program on European adolescents’ patterns of alcohol use. J Adolesc Health. 2011;48(2):182–8. 21257118 10.1016/j.jadohealth.2010.06.003

[60] Sanchez ZM, Valente JY, Galvão PP, Gubert FA, Melo MHS, Caetano SC, et al. A cluster randomized controlled trial evaluating the effectiveness of the school-based drug prevention program #Tamojunto2.0. Addiction. 2021;116(6):1580–92. 33245788 10.1111/add.15358

[61] Graham JW, Marks G, Hansen WB. Social influence processes affecting adolescent substance use. J Appl Psychol. 1991;76(2):291–8. 2055870 10.1037/0021-9010.76.2.291

[62] Domitrovich CE, Bradshaw CP, Poduska JM, Hoagwood K, Buckley JA, Olin S, et al. Maximizing the implementation quality of evidence-based preventive interventions in schools: a conceptual framework. Adv Sch Ment Health Promot. 2008;1(3):6–28. 27182282 10.1080/1754730x.2008.9715730 PMC4865398

[63] Rohrbach LA, Ringwalt CL, Ennett ST, Vincus AA. Factors associated with adoption of evidence-based substance use prevention curricula in US school districts. Health Educ Res. 2005;20(5):514–26. 15687101 10.1093/her/cyh008

[64] OECD. How Much Time Do Teachers Spend on Teaching and Non-teaching Activities? Education Indicators In Focus, 29: OECD; 2015. Available from: https://www.oecd.org/en/publications/how-much-time-do-teachers-spend-on-teaching-and-non-teaching-activities_5js64kndz1f3-en.html.

[65] Strein W, Kuhn-McKearin MN, Finney M. Best practices in developing prevention strategies for school psychology practice. 2007.

[66] Yoder N, Ward AM, Wolforth JD. Instructional practices that integrate equity-centered social, emotional, and academic learning. American Institutes for Research; 2021. Available from: https://www.air.org/sites/default/files/2021-12/Social-Emotional-Learning-Equity-Centered-Instructional-Practices-December-2021.pdf.

[67] Darling-Hammond L, Flook L, Cook-Harvey C, Barron B, Osher D. Implications for educational practice of the science of learning and development. Appl Dev Sci. 2020;24(2):97–140.

[68] OECD. OECD Indicators. Paris: OECD Publishing; 2022. p. 2022.

[69] Ministerio de Educación de Chile. Base de datos Matrícula Oficial 2023 Santiago, Chile: Ministerio de Educación; 2023. Available from: https://centroestudios.mineduc.cl/2023/11/02/base-de-datos-matricula-oficial-2023-disponible/.

