# Supplementary material for: Acceptability, feasibility, fidelity and quality implementation of the culturally adapted version of the Social Competence Promotion Program among Young Adolescents (“Mi Mejor Plan”) to prevent substance use among adolescents in Chile: a pilot randomized control study
Source: BMC Public Health. 2025 May 20;25:1860. doi: 10.1186/s12889-025-23033-3 (PMC12090675; doi:10.1186/s12889-025-23033-3)
Supplement: Supplementary file 1 — Supplementary Material 1. [file 12889_2025_23033_MOESM1_ESM.docx]

Supplement 1: Content adaptation to the Chilean context of SCPP-YA Program.

| **SCPP-YA** | **Adaptation** |
| --- | --- |
| ***Social problem-solving module*** | |
| **Session 1: Presentation of Social Problem Solving** | |
| 1. Presentation of the Resolution of Social Problems and the activity "All About Me". 2. Students interview each other using Student Worksheet number 1 “All About Me”. 3. Students share with the class two interesting facts about their interviewee. 4. Students share something that makes them feel upset or stressed. 5. Students read the steps on Wall Poster number 2 “Problem Solving Traffic Light Poster”. 6. Students receive a program description letter for their parents. | Stays the same as the original. It is session number 1 in the adapted version (MMP) and has three main activities called “All About Me”, “Introduction to Problem Solving method” and “Introduction to the letter for parents”. |
| **Session 2: Adaptively Responding to Stress and Problems** | |
| 1. Review of Student Worksheet number 1 “All About Me” and parent feedback on the program. 2. Presentation of concepts about stress. 3. Reactions of the students to the Stressful Situations Cards. 4. Introduction and presentation of a Stressful Situation. 5. Discussion about a Stressful Situation and adaptive ways to manage stress. | Stays the same as the original. It is session number 2 in the adapted version (MMP) and has three main activities called “Presentation of concepts about stress”, “Stressful Situations Cards” and “Stressful Situations Comic”. |
| **Session 3: Benefits of Staying Calm in Stressful Situations** | |
| 1. Students review the steps on the Problem-Solving Traffic Light Poster. 2. The teacher talks about the use of the steps, in real everyday situations. 3. Students describe times when they became angry or lost control. 4. Students give reasons to stop and calm down. 5. How to Save the Program material. | Stays the same as the original but also including sessions 4 and 5. It is session number 3 in the adapted version (MMP) and has four main activities called “Problem-Solving Traffic Light Poster”, “Problem-Solving in real everyday situations”, “Introduction to the first step in Problem-Solving: Stop, calm down, and think before you act” and “Learning how to calm down” |
| **Session 4: Learn a Way to Calm Down** | |
| 1. Students talk about their observations and about how people calm down when they are stressed. 2. Students learn and practice a calming breathing technique. 3. Students complete Student Worksheet number 3: “Problem Log 1”. 4. Students share what they wrote on Student Worksheet number 3: “Problem Log 1”. 5. Students develop a code that summarizes the concepts learned to date. | Included in session number 3 in the adapted version (MMP). |
| **Session 5: More Ways to Calm Down and Manage Stress** | |
| 1. Students read the results of the code and review the first step and the breathing technique. 2. The teacher presents “the Stress Thermometer”. 3. Students work in small groups to respond to stressful situations, listed on cards, in ways that could calm them down. 4. Groups of students share with the class. 5. The teacher presents the discussion activity about the Problem-Solving method. 6. Students play a game to review the concepts learned. | Included in session number 3 in the adapted version (MMP). |
| **Session 6: Identifying and Sharing Feelings** | |
| 1. The teacher introduces the second step of the problem-solving method. 2. Students associate feelings with situations written on cards. 3. The teacher introduces the “Dictionary of Feelings” and the concept of looking, listening, or asking about feelings. 4. Students search for pictures, words, or actions to identify feelings. 5. The “Parent Involvement Letter Number 2” is given to the students. | Stays the same as the original. It is session number 4 in the adapted version (MMP) and has four main activities called “Introduction to the step two in Problem-Solving: Say the problem and how you feel”, Associate feelings with situations”, “Dictionary of Feelings” and “Parent Involvement Letter”. |
| **Session 7: Associating Feelings and Situations** | |
| 1. Students add new words to the “Feelings Dictionary” and share their parents' reactions to “Parent Involvement Letter number 2”. 2. 2. Students complete a worksheet for student: Feelings and Situations, which allows visualizing the complexity of feelings and situations. 3. Students role-play about the problems in the workbook. 4. Discussion about the problem-solving method. | Stays the same as the original. It is session number 5 in the adapted version (MMP) and has two main activities called “Feelings and Situations” and “Discussion about the problem-solving method”. |
| **Session 8: Common Problems in Adolescents** | |
| 1. The teacher introduces what it is to define a problem. 2. Students work in groups to identify problem situations that are important to them. 3. Students share problem situations with the class. 4. Students brainstorm and then complete a worksheet that examines the importance of “Find the problem and say how I feel”. | Stays the same as the original. It is session number 6 in the adapted version (MMP) and has three main activities called “Define a problem”, “Students identify problem situations that are important to them” and “Students share problem situations with the class”. |
| **Session 9: Role Play for the first two steps of Problem-Solving** | |
| 1. Discussion about the problem-solving method. 2. The teacher and two students role-play and discuss a problem situation. 3. Students apply step 1 and 2 to three problem situations. 4. Students play a game of challenges to review the concepts learned thus far. | Session deleted. |
| **Session 10: The Importance of Setting Positive Goals** | |
| 1. The teacher reviews the steps of the problem-solving method and uses the worksheet to introduce the concept of goal. 2. Students complete a worksheet to practice identifying positive goals. 3. Students share the answers on the worksheet and check that they are correct. 4. Students discuss situations where they did not have a positive goal. 5. Students work in pairs to complete the worksheet in which they practice identifying feelings or emotions and generating positive goals. | Stays the same as the original. It is session number 7 in the adapted version (MMP) and has 3 main activities called “Introduction to the step three in Problem-Solving: Think of a positive goal”, “Students practice identifying positive goals” and “Students practice generating positive goals”. |
| **Session 11: Applying the First Three Steps of the Problem- Solving Method** | |
| 1. Students work in class to create a list of ways to recognize when goals are positive. 2. Students share their work from Student Worksheet 12: Giving a Positive Emotion and Target! and review the worksheet against the list of ways to recognize when goals are positive. 3. Students use a role play to practice the first three steps of the problem-solving method. 4. Students are given the "Problem Record number 2" | Session deleted. |
| **Session 12: Looking for Solutions to Problems** | |
| 1. Students discuss Student Worksheet 13: Problem Log 2. 2. The concept of “solution” is introduced to the students and the fourth step of the problem-solving method. 3. Students complete and discuss Student Worksheet 14: “Making New Friends”. 4. Students complete and discuss Student Worksheet 15 “Helping Mom Feel Better”. | Session deleted. |
| **Session 13: Working Together to Find Solutions** | |
| 1. Discussion about the problem-solving method. 2. Students work in groups to find many solutions in the Solution Finding Contest. 3. Students receive diplomas for generating many solutions. 4. Students describe what they have learned by looking for many solutions. | Students do not receive diplomas.  Session 14 was included. It is session number 8 in the adapted version (MMP) and has three main activities called “Introduction to the step four in Problem-Solving: Think of lots of solutions”, “Solution Finding Contest” and “Students practice the first four steps of the problem-solving method”. |
| **Session 14: Practicing the First Four Steps of the Problem-Solving Method** | |
| 1. The teacher presents the comic "Nicolas's problem". 2. Students identify Nicolás's problems from the comic “Nicolas's Problem”. 3. The class works using student worksheet number 16 “Nicolas' Problem” to practice the first four steps of the problem-solving method for some of Nicholas's problems. 4. Students are given Homework: Student Worksheet Number 17: Problem Record Number 3. | Included in session number 8 in the adapted version (MMP). |
| **Session 15: What Could Happen Next?** | |
| 1. Students discuss Student Worksheet number 17: Problem Log number 3. 2. The teacher introduces step 5 of the problem-solving method and the concept of consequences. 3. Students work in pairs to complete Student Worksheet 19 “What Might Happen Next?”. 4. Students share the results with the class, checking that the anticipations of the consequences are realistic. 5. The teacher shares a problem with the class and the students apply the first five steps of the problem-solving method to it. | Stays the same as the original. It is session number 9 in the adapted version (MMP) and has four main activities called “Introduction to the step five in Problem-Solving: Think ahead to the consequences”, “Realistic consequences” and “Students apply the first five steps of the problem-solving method”. |
| **Session 16: Uniting Solutions and Consequences** | |
| 1. The teacher presents the worksheet for the student number 20 “Thinking in Solutions and their Consequences”. 2. Students work in groups of 3 or 4 to complete Student Worksheet number 20 “Thinking in Solutions and Consequences”. 3. Students share and discuss the results of their work, reviewing the evaluation of possible solutions. 4. Students complete two review sheets to test their knowledge. | Session deleted. |
| **Session 17: Why Didn't the Solution Work?** | |
| 1. The teacher reviews the answers to student worksheet number 21: “Stop-Think-Go” Review Guide. 2. Students read a story about a problem and then work in groups to discover why the solution didn't work. 3. Groups share and think of ways to improve the way solutions are carried out. 4. The teacher presents Step 6 of the Problem-Solving Method: Planning and the elements of a good plan. | Session 18 was included (Activities 1-3). It is session number 10 in the adapted version (MMP) and has five main activities called “Introduction to the step six in Problem-Solving: Go ahead and try the best plan using interactive methodologies”, “Story about a problem”, “Ways to improve the way solutions are carried out using the six steps”, “Tones of voice” and “Body language” |
| **Session 18: Moment, Tone of Voice, and Body Language** | |
| 1. The teacher reviews the elements of a good plan. 2. Students compare the use of hostile, passive, and cooperative tones of voice. 3. Students compare the use of hostile, passive, and cooperative body language. 4. Students work in pairs to complete “Plan Ahead” Student Worksheet number 24. 5. Discussion of Student Worksheet number 24 “Plan Ahead”. | Included in sessions number 10 and 11 in the adapted version (MMP). |
| **Session 19: Patience and Perseverance Help in Problem Solving** | |
| 1. Students try to untie "The Human Knot". 2. Student volunteers role-play a situation that shows Perseverance. 3. Students apply the six steps of the Problem-Solving Method to life situations. | The activity “The Human Knot” was deleted.  Session 18 was included (Activities 4-5). It is session number 11 in the adapted version (MMP) and has three main activities called “Students work in pairs to complete Plan Ahead Student Worksheet”, “Students discuss the Plan Ahead Student Worksheet”, and “Role-play a situation that shows Perseverance”. |
| **Session 20: Applying Problem Solving to make the world a better place** | |
| 1. Students discuss Student Worksheet number 25 “Becoming a Successful Problem Solver”. 2. Students act out two problem situations. 3. The teacher introduces “101 ways to make this world a better place”, and the letter to parents. | Session deleted. |
| **Session 21: Recognizing Resources** | |
| 1. Discussion about problem-solving “101 ways to make this world a better place”. 2. The teacher introduces the question of who you turn to when you want to discuss a problem. 3. Students generate a list of people they can turn to for help with their problems. 4. The group discusses the people they turn to when they have a difficult situation. 5. Students generate personal and group problems. | Session deleted. |
| **Session 22: Solving a Group Problem** | |
| 1. Discussion about problem-solving “101 ways to make this world a better place”. 2. Students solve a group problem. 3. Students solve a school or community problem | Session deleted. |
| **Session 23: Applying Problem Solving in Relationships** | |
| 1. Discussion about problem-solving “101 ways to make this world a better place”. 2. Introduction of the comic "Friends". 3. Students discuss the comic “Friends” using the point of view of two of the main characters. | Session deleted. |
| **Session 24: Review of the Problem-Solving Method – Trivia Game** | |
| 1. Discussion about problem-solving method “101 ways to make this world a better place”. 2. The teacher introduces the Problem-Solving Trivia Game and organizes the students into teams. 3. Students play the Problem-Solving Trivia Game. 4. The class talks about what they learned playing the game. | Session deleted. |
| **Session 25: Creation of role plays for the Problem-Solving method** | |
| 1. Discussion about problem-solving method “101 ways to make this world a better place”. 2. The teacher introduces the role play creation activity. 3. Students prepare the role plays in groups. | Session deleted. |
| **Session 26: Role playing of the Problem-Solving method** | |
| 1. Students add examples to the “101 ways to make this world a better place” list. 2. Students form groups to act out and discuss situations | Session deleted. |
| **Session 27: Reflecting on the Problem-Solving method** | |
| 1. Students complete a questionnaire about the program. 2. Students review their folders and the list of “101 ways to make this world a better place”. 3. Students discuss the Problem-Solving program. 4. Students receive a diploma certifying the successful completion of the Problem-Solving Program. | Session deleted. |
| ***The substance use prevention module*** | |
| **Session 1: Establishing Goals for Healthy Life Styles** | |
| 1. Students discuss the transition from the Problem-Solving Unit to the Drug Use Unit. 2. Learning the term goals. 3. Setting personal goals. 4. Role models. 5. The task Interviewing Someone You Admire is stated. | Stays the same as the original. It is session number 12 in the adapted version (MMP) and has three main activities called “Learning the term goals”, “Setting personal goals” and “Role models”. |
| **Session 2: Tobacco and Alcohol: Myths and Realities** | |
| 1. Discussion of the task: Interview someone I admire. 2. Myths and realities-team play. 3. Review of Realities on the use of Tobacco and Alcohol. 4. Evaluating Advertisements, tv-series, and Cinema regarding the use of Cigarettes and Alcohol. 5. Presentation of the task, Luisa's Reasons. | Stays the same as the original. It is session number 13 in the adapted version (MMP) and has two main activities called “Myths and realities-team play” and “Review of Realities on the use of Tobacco and Alcohol”. |
| **Session 3: What do we know about drugs? Information about Marijuana, Cocaine and Crack** | |
| 1. What do you know about tobacco, alcohol, and other drugs? 2. Analyzing the consequences of drug use. 3. Students reflect on their Journal entries. 4. Presentation of the Task: Interview with Parents/Mothers. | Stays the same as the original. It is session number 14 in the adapted version (MMP) and has two main activities called What do you know about tobacco, alcohol, and other drugs?” and “Consequences of drug use”. |
| **Session 4: Peer influences** | |
| 1. Reviewing the Parent Letter. 2. Understanding Peer Pressure. 3. Discussing Ways to Say No. 4. Presentation of the task: Keeping a personal diary | Stays the same as the original. It is session number 15 in the adapted version (MMP) and has two main activities called “Understanding Peer Pressure” and “Discussing Ways to Say No”. |
| **Session 5: Twelve ways to say no. Standing up to peer pressure** | |
| 1. Reviewing the Life Diary Entry. 2. Techniques to say No. 3. Role Playing for Effective Strategies | Stays the same as the original, a closure to the program was included. It is session number 16 in the adapted version (MMP) and has three main activities called “Techniques to say No”, “Role Playing for Effective Strategies” and “Program closure” |
| **Session 6: Getting help – Knowing when to tell** | |
| 1. Discussion of a videotape about drug abuse. 2. Discussion of resources for help | Session deleted. |
| **Session 7: Ask the experts – A policeman, school social worker and drug abuse counselor discuss their views on substance use** | |
| 1. Speakers’ presentations 2. Question and answer period 3. Summarizing comments | Session deleted. |
| **Session 8: Teaching others about drug abuse prevention (Community/School awareness project): Part I** | |
| 1. Jeopardy game 2. Write a substance use prevention project | Session deleted. |
| **Session 9: Teaching others about drug abuse prevention (Community/School awareness project): Part II** | |
| 1. Presentation of the substance use prevention project | Session deleted. |
