# Supplementary material for: Acceptability, feasibility, fidelity and quality implementation of the culturally adapted version of the Social Competence Promotion Program among Young Adolescents (“Mi Mejor Plan”) to prevent substance use among adolescents in Chile: a pilot randomized control study
Source: BMC Public Health. 2025 May 20;25:1860. doi: 10.1186/s12889-025-23033-3 (PMC12090675; doi:10.1186/s12889-025-23033-3)
Supplement: Supplementary file 2 — Supplementary Material 2. [file 12889_2025_23033_MOESM2_ESM.docx]

Supplement 2. Adherence and Pedagogy by Observers’ Reports.

|  |  | Adherence^1^ | | | | Pedagogy^3^ |
| --- | --- | --- | --- | --- | --- | --- |
| Observation Number | Session  number | Opening  % | Central  % | Closure  % | Content knowledge^2^  % | Group Management  % |
| 1 | 1 | 80 | 100 | 100 | 80 | 60 |
| 2 | 1 | 100 | 80 | 100 | 80 | 80 |
| 3 | 1 | 100 | 100 | 100 | 100 | 80 |
| 4 | 2 | 80 | 80 | 100 | 100 | 100 |
| 5 | 2 | 100 | 100 | 100 | 100 | 100 |
| 6 | 2 | 100 | 100 | 80 | 100 | 100 |
| 7 | 2 | 100 | 100 | 100 | 100 | 100 |
| 8 | 3 | 100 | 60 | 60 | 100 | 100 |
| 9 | 3 | 100 | 100 | 100 | 100 | 80 |
| 10 | 3 | 100 | 20 | 100 | 100 | 60 |
| 11 | 4 | 100 | 100 | 100 | 100 | 100 |
| 12 | 4 | 100 | 100 | 100 | 100 | 100 |
| 13 | 4 | 100 | 80 | 100 | 100 | 100 |
| 14 | 4 | 100 | 80 | 100 | 100 | 100 |
| 15 | 5 | 80 | 80 | 100 | 100 | 80 |
| 16 | 5 | 100 | 100 | 100 | 100 | 100 |
| 17 | 5 | 100 | 100 | 100 | 100 | 100 |
| 18 | 5 | 60 | 80 | 80 | 100 | 60 |
| 19 | 5 | 100 | 100 | 0 | 100 | 100 |
| 20 | 6 | 100 | 80 | 20 | 100 | 60 |
| 21 | 6 | 100 | 80 | 0 | 100 | 80 |
| 22 | 6 | 100 | 100 | 100 | 100 | 80 |
| 23 | 7 | 100 | 80 | 100 | 100 | 100 |
| 24 | 7 | 100 | 60 | 0 | 100 | 80 |
| 25 | 7 | 100 | 60 | 100 | 80 | 100 |
| 26 | 8 | 100 | 80 | 100 | 100 | 100 |
| 27 | 8 | 100 | 60 | 0 | 100 | 100 |
| 28 | 8 | 100 | 60 | 100 | 100 | 100 |
| 29 | 9 | 100 | 100 | 100 | 100 | 100 |
| 30 | 9 | 100 | 60 | 100 | 100 | 80 |
| 31 | 10 | 100 | 100 | 100 | 100 | 100 |
| 32 | 10 | 100 | 100 | 100 | 100 | 100 |
| 33 | 10 | 100 | 80 | 0 | 100 | 100 |
| 34 | 11 | 100 | 80 | 40 | 100 | 60 |
| 35 | 11 | 100 | 80 | 100 | 100 | 60 |
| 36 | 11 | 100 | 80 | 100 | 100 | 100 |
| 37 | 12 | 100 | 100 | 100 | 100 | 100 |
| 38 | 12 | 100 | 100 | 100 | 100 | 100 |
| 39 | 13 | 100 | 20 | 100 | 100 | 100 |
| 40 | 13 | 100 | 20 | 100 | 100 | 100 |
| 41 | 13 | 100 | 40 | 80 | 100 | 100 |
| 42 | 13 | 100 | 40 | 0 | 100 | 100 |
| 43 | 14 | 100 | 80 | 100 | 100 | 80 |
| 44 | 14 | 80 | 100 | 100 | 100 | 100 |
| 45 | 14 | 60 | 40 | 60 | 100 | 60 |
| 46 | 15 | 100 | 60 | 100 | 100 | 80 |
| 47 | 15 | 0 | 80 | 100 | 100 | 100 |
| 48 | 15 | 100 | 60 | 100 | 100 | 100 |
| 49 | 15 | 80 | 80 | 0 | 100 | 100 |
| 50 | 16 | 100 | 100 | 100 | 100 | 100 |
| 51 | 16 | 100 | 80 | 100 | 80 | 100 |
| 52 | 16 | 100 | 80 | 100 | 100 | 80 |
| Total mean | - | 94.6 | 78.5 | 81.2 | 98.5 | 90.4 |

Notes:

^1^ The assessment was based on the following scoring in a 6-level scale according to the degree of task or activity completeness: (1) Not Started, 0% (The task or activity has not begun; No progress has been made.); (2) Barely Started, 20% (Minimal progress has been achieved; Only a small portion of the task has been addressed); (3) Partially Complete, 40% (The task is underway, but less than half is complete; Some components are in progress, but significant parts remain untouched); (4) Moderately Complete, 60% (More than half of the task is complete; Most major components are in progress, but the task is far from finished.); (5)Almost Complete, 80% (The task is nearing completion; Final details or minor adjustments remain.); (6) Fully Complete, 100% (The task or activity is entirely finished; All objectives have been met with no pending work.)

^2^ Regarding the degree of knowledge of the manual demonstrated by the facilitator, the assessment was based on the following scoring in a 6-level scale: (1) No Knowledge, 0% (The manual has not been reviewed. Complete lack of awareness of its content and structure.); (2) Basic Initial Knowledge, 20% (The manual has been briefly skimmed. General structure is recognized, but details and practical applications are not understood.); (3) Partial Knowledge, 40% (Some parts of the manual have been reviewed with a basic understanding. Certain sections are clear, but key elements necessary for conducting a proper session are missing.); (4) Moderate Knowledge, 60% (Most of the manual’s content is understood. There is enough confidence to present a session with the manual’s support, though minor doubts or errors may arise.); (5) Advanced Knowledge, 80% (Nearly all of the manual’s content is mastered. Sessions can be presented smoothly and in alignment with the manual, with minimal uncertainties.); (6) Expert Knowledge, 100% (The manual is fully mastered. Sessions can be delivered accurately, flexibly, and without consulting the manual during execution.)

^3^ Regarding the group management scale for classroom facilitation, the assessment was based on the following scoring in a 6-level scale: (1) No Control, 0% (The facilitator struggles to maintain basic order and discipline. Students are disengaged, disruptive, or inattentive, and the session cannot proceed as planned.); (2) Minimal Control, 20% (The facilitator can occasionally redirect attention but has difficulty managing disruptions. The session progresses with significant interruptions, and only a small portion of the group remains focused.); (3) Partial Control, 40% (The facilitator demonstrates some ability to manage the group. Attention and participation fluctuate, and the session continues with periodic distractions.); (4) Moderate Control, 60% (The facilitator maintains general order and engagement. Most students participate, but occasional minor disruptions or lapses in attention occur.); (5) Strong Control, 80% (The facilitator effectively manages the group with confidence and authority. Students are attentive, engaged, and disruptions are minimal and quickly addressed.); (6) Mastery of Group Management, 100% (The facilitator fully commands the group with ease and adaptability. The session flows seamlessly, with students actively participating and staying focused throughout.)
