# Supplementary material for: Acceptability, feasibility, fidelity and quality implementation of the culturally adapted version of the Social Competence Promotion Program among Young Adolescents (“Mi Mejor Plan”) to prevent substance use among adolescents in Chile: a pilot randomized control study
Source: BMC Public Health. 2025 May 20;25:1860. doi: 10.1186/s12889-025-23033-3 (PMC12090675; doi:10.1186/s12889-025-23033-3)
Supplement: Supplementary file 3 — Supplementary Material 3. [file 12889_2025_23033_MOESM3_ESM.docx]

Supplement 3: Fidelity as reported by the Facilitator.

Table 1: Sessions and adherence to the manual by the facilitator.

|  | N total (Total number of sessions per facilitator) | I implemented the session in its entirety  (n = 99) | | I did not implement the session in its entirety due to lack of time  (n = 48) | | I did not implement the session in its entirety  due to classroom climate  (n = 13) | |
| --- | --- | --- | --- | --- | --- | --- | --- |
|  | n | n | % | n | % | n | % |
| Facilitator 1 | 86 | 43 | 50.0 | 37 | 43.0 | 6 | 7.0 |
| Facilitator 2 | 31 | 17 | 54.8 | 7 | 22.6 | 7 | 22.6 |
| Facilitator 3 | 26 | 24 | 92.3 | 2 | 7.7 | 0 | 0 |
| Facilitator 4 | 8 | 8 | 100 | 0 | 0 | 0 | 0 |
| Facilitator 5 | 5 | 3 | 60.0 | 2 | 40.0 | 0 | 0 |
| Facilitator 6 | 4 | 4 | 100 | 0 | 0 | 0 | 0 |

Table 2: Sessions and adherence to the manual by school.

|  | N total (Total number of sessions per school) | I implemented the session in its entirety  (n = 99) | | I did not implement the session in its entirety due to lack of time  (n = 48) | | I did not implement the session in its entirety  due to classroom climate  (n = 13) | |
| --- | --- | --- | --- | --- | --- | --- | --- |
|  | n | n | % | n | % | n | % |
| School 1 | 32 | 19 | 59.4 | 9 | 28.1 | 4 | 12.5 |
| School 2 | 32 | 20 | 62.5 | 11 | 34.4 | 1 | 3.1 |
| School 3 | 32 | 13 | 40.6 | 19 | 59.4 | 0 | 0 |
| School 4 | 32 | 27 | 84.4 | 5 | 15.6 | 0 | 0 |
| School 5 | 32 | 20 | 62.5 | 4 | 12.5 | 8 | 25.0 |
